# Comparative Evaluation of AI Models Such as ChatGPT 3.5, ChatGPT 4.0, and Google Gemini in Neuroradiology Diagnostics

**DOI:** 10.7759/cureus.67766

**Published:** 2024-08-25

**Authors:** Rishi Gupta, Abdullgabbar M Hamid, Miral Jhaveri, Niki Patel, Pokhraj P Suthar

**Affiliations:** 1 Department of Diagnostic Radiology and Nuclear Medicine, Rush University Medical Center, Chicago, USA; 2 Department of Osteopathic Medicine, Kentucky College of Osteopathic Medicine, Pikeville, USA

**Keywords:** chatgpt 3.5, neuroradiology, google gemini, chatgpt 4, ai

## Abstract

Aims and objective: Advances in artificial intelligence (AI), particularly in large language models (LLMs) like ChatGPT (versions 3.5 and 4.0) and Google Gemini, are transforming healthcare. This study explores the performance of these AI models in solving diagnostic quizzes from "Neuroradiology: A Core Review" to evaluate their potential as diagnostic tools in radiology.

Materials and methods: We assessed the accuracy of ChatGPT 3.5, ChatGPT 4.0, and Google Gemini using 262 multiple-choice questions covering brain, head and neck, spine, and non-interpretive skills. Each AI tool provided answers and explanations, which were compared to textbook answers. The analysis followed the STARD (Standards for Reporting of Diagnostic Accuracy Studies) guidelines, and accuracy was calculated for each AI tool and subgroup.

Results: ChatGPT 4.0 achieved the highest overall accuracy at 64.89%, outperforming ChatGPT 3.5 (62.60%) and Google Gemini (55.73%). ChatGPT 4.0 excelled in brain, head, and neck diagnostics, while Google Gemini performed best in head and neck but lagged in other areas. ChatGPT 3.5 showed consistent performance across all subgroups.

Conclusion:This study found that advanced AI models, including ChatGPT 4.0 and Google Gemini, vary in diagnostic accuracy, with ChatGPT 4.0 leading at 64.89% overall. While these tools are promising in improving diagnostics and medical education, their effectiveness varies by area, and Google Gemini performs unevenly across different categories. The study underscores the need for ongoing improvements and broader evaluation to address ethical concerns and optimize AI use in patient care.

## Introduction

Advancements in artificial intelligence (AI), particularly in the realm of large language models (LLMs), are ushering in a new era in healthcare [1]. Among these LLMs, ChatGPT (versions 3.5 and 4.0) and Google Gemini stand out as widely used and accessible models [2,3]. ChatGPT 4.0, in particular, represents a significant leap forward, having been trained on an extensive dataset that includes radiology articles as part of the GPT-4 training process [4]. This innovative integration has sparked a wave of research exploring the potential applications of ChatGPT in assessment and educational contexts. One of the most promising areas of exploration is the evaluation of ChatGPT (3.5 and 4.0) and Google Gemini in solving diagnostic quizzes and exams [2]. These studies reflect the broader potential of LLMs to enhance medical diagnostics and patient care and revolutionize medical education and examination systems [5]. This research aims to ascertain the precision of ChatGPT (3.5 and 4.0) and Google Gemini in solving diagnostic quizzes from the textbook "Neuroradiology: A Core Review." By doing so, we aim to evaluate their potential as supportive tools in radiological diagnostics and their unique contributions to the existing body of knowledge. Measuring the performance of ChatGPT (3.5 and 4.0) and Google Gemini in these complex clinical scenarios will allow us to explore their potential as decision-support tools in diagnostic radiology. This analysis is an essential step toward incorporating AI-driven models into healthcare, thereby improving medical professionals' decision-making and potentially enhancing patient outcomes.

## Materials and methods

The primary objective of this study was to determine the accuracy of ChatGPT 3.5 (version 3.5-turbo), ChatGPT 4.0 (version 4.0-turbo), and Google Gemini (version 1.0) in solving diagnostic quizzes derived from the textbook "Neuroradiology: A Core Review" by Dubey et al. [6]. The focus was on evaluating the accuracy of these AI tools when provided with quiz questions on the brain, head and neck, spine, and non-interpretive skill parts of the nervous system. This study was based on publicly accessible literature, so there was no requirement for obtaining ethical approval. The research design strictly adhered to the Standards for Reporting Diagnostic Accuracy (STARD) guidelines, ensuring that our methodology met the highest standards of accuracy and transparency in diagnostic research. By adhering to these guidelines, we aimed to maintain rigorous scientific integrity and reliability throughout our study.

Given the inability of ChatGPT 3.5 and Google Gemini to process images, we excluded questions that relied solely on images. Ultimately, we included 262 multiple-choice cases in our analysis. Each case comprised a text prompt along with four potential choices (a, b, c, and d).

**Figure 1:**
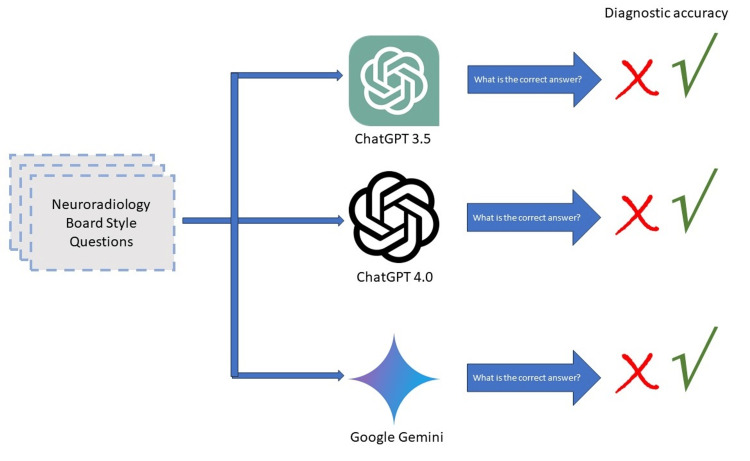
A visual summary of the study This study assesses the diagnostic accuracy of ChatGPT 3.5, ChatGPT 4.0, and Google Gemini in answering neuroradiology board-style questions. The questions are sourced from the textbook "Neuroradiology: A Core Review" by Dubey et al. [6]. The evaluation aims to compare the performance of these AI models in solving complex neuroradiology scenarios.

The 262 multiple-choice questions were systematically fed into three AI tools: ChatGPT 3.5, ChatGPT 4.0, and Google Gemini. Each AI tool was prompted with the same question: "What is the correct answer?" The responses from each AI tool included an answer choice and an explanation for their selection. Figure 1 provides a visual summary of the study, illustrating the workflow and categorization of cases.

Independent radiologists reviewed and confirmed the alignment of the diagnoses generated by each AI tool with those previously published in the textbook. This verification process ensured the accuracy and reliability of the results. The AI tools’ responses were then compared to the answer key to determine their correctness.

The responses to the 262 cases from all three tools were compiled to calculate the accuracy of each respective tool. All relevant data were recorded in a Microsoft Excel spreadsheet (Microsoft Corp., Redmond, Washington), enabling a comprehensive evaluation of accuracy as a percentage. Additionally, the cases were categorized into four subgroups: brain, head and neck, spine, and non-interpretive skills. Separate analyses were conducted for each subtype to assess the accuracy of ChatGPT 3.5, ChatGPT 4.0, and Google Gemini within these domains. A visual summary of the study, including the workflow, categorization of cases, and accuracy percentages of each AI tool across different subgroups, is shown in Figure 1. This visualization provides a clear overview of the study design and the performance of the AI tools.

All the relevant data were compiled in a Microsoft Excel spreadsheet. To ensure the robustness of our findings, we performed statistical analysis on the collected data. We calculated the accuracy of each AI tool as a percentage of correct responses out of the total number of cases. Additionally, we conducted subgroup analyses to determine the performance of each AI tool within the different categories (brain, head and neck, spine, and non-interpretive skills). Comparative analyses were performed to highlight differences in performance among ChatGPT 3.5, ChatGPT 4.0, and Google Gemini.

## Results

**Figure 2:**
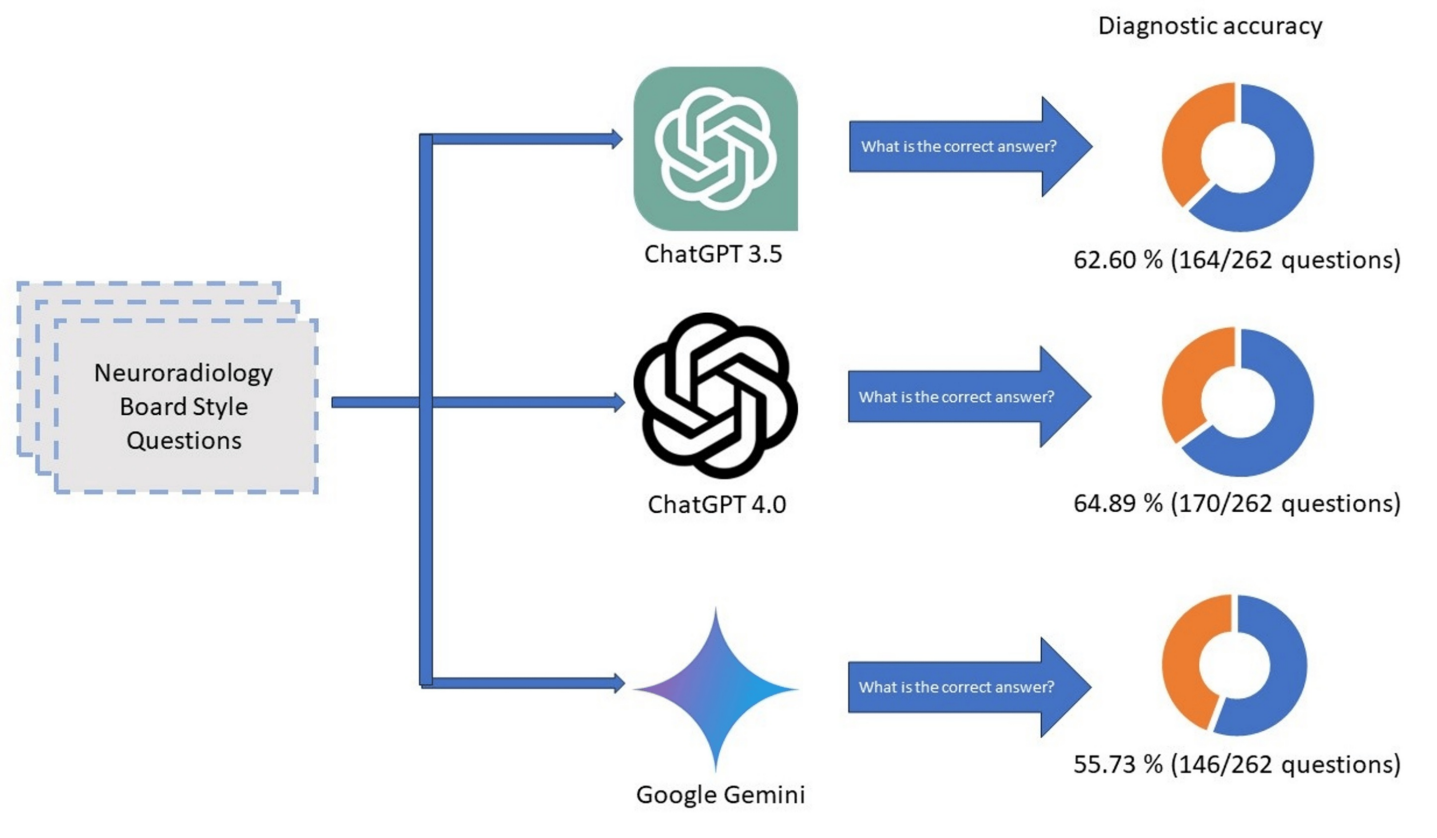
This figure illustrates the diagnostic accuracy of ChatGPT 4.0, ChatGPT 3.5, and Google Gemini in solving neuroradiology questions. ChatGPT 4.0 achieved the highest overall diagnostic accuracy at 64.89% compared to ChatGPT 3.5 at 62.60% and Google Gemini at 55.73%.

The diagnostic performance of ChatGPT 3.5, ChatGPT 4.0, and Google Gemini varied significantly. The overall diagnostic accuracy was 64.89% for ChatGPT 4.0 (170 out of 262 cases), 62.60% for ChatGPT 3.5 (164 out of 262 cases), and 55.73% for Google Gemini (146 out of 262 cases). The accuracy metrics are shown in Figure 2.

**Figure 3:**
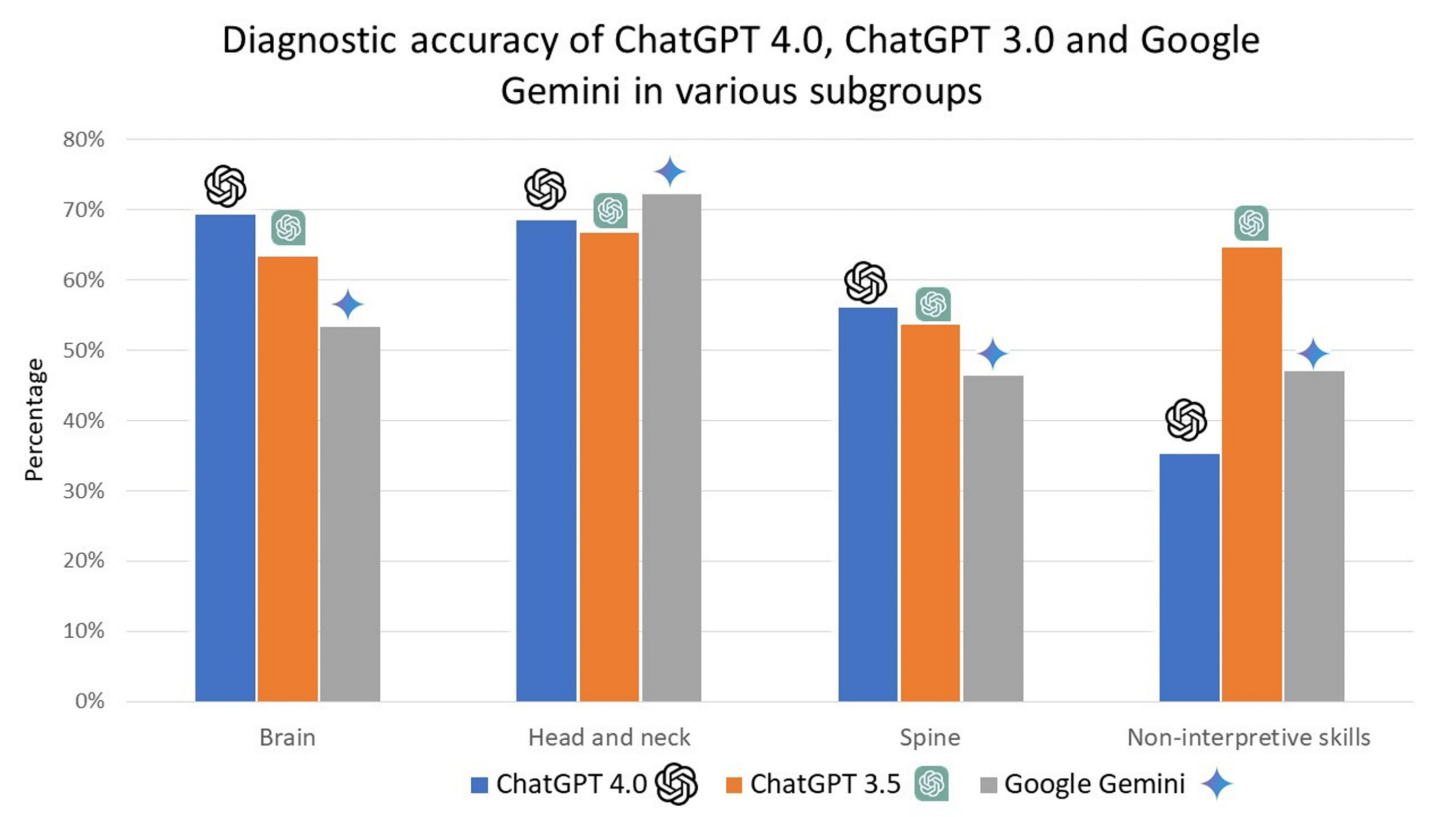
This figure presents the diagnostic accuracy of ChatGPT 3.5, ChatGPT 4.0, and Google Gemini across different neuroradiology subgroups.

The comprehensive diagnostic proficiency of ChatGPT (versions 3.5 and 4.0) and Google Gemini varied across different subgroups of the nervous system. For ChatGPT 4.0, the diagnostic accuracy was 69.33% (104 out of 150 cases) for brain-related questions, 68.52% (37 out of 54 cases) for head and neck, 56.10% (23 out of 41 cases) for spine, and 52.94% (nine out of 17 cases) for non-interpretive skills related to the nervous system. ChatGPT 4.0's performance was particularly notable in the brain and head and neck subgroups, indicating a robust capability in these areas. For ChatGPT 3.5, the diagnostic accuracy was 63.33% (95 out of 150 cases) for brain-related questions, 66.67% (36 out of 54 cases) for head and neck, 53.66% (22 out of 41 cases) for spine, and 64.71% (11 out of 17 cases) for non-interpretive skills related to the nervous system. While slightly lower than ChatGPT 4.0, ChatGPT 3.5 demonstrated consistent performance across the various subgroups. For Google Gemini, the diagnostic accuracy was 53.33% (80 out of 150 cases) for brain-related questions, 72.22% (39 out of 54 cases) for head and neck, 46.34% (19 out of 41 cases) for spine, and 47.06% (eight out of 17 cases) for non-interpretive skills related to the nervous system (Figure 3). Google Gemini showed its highest performance in the head and neck subgroup but lagged in other areas compared to the ChatGPT versions.

**Figure 4:**
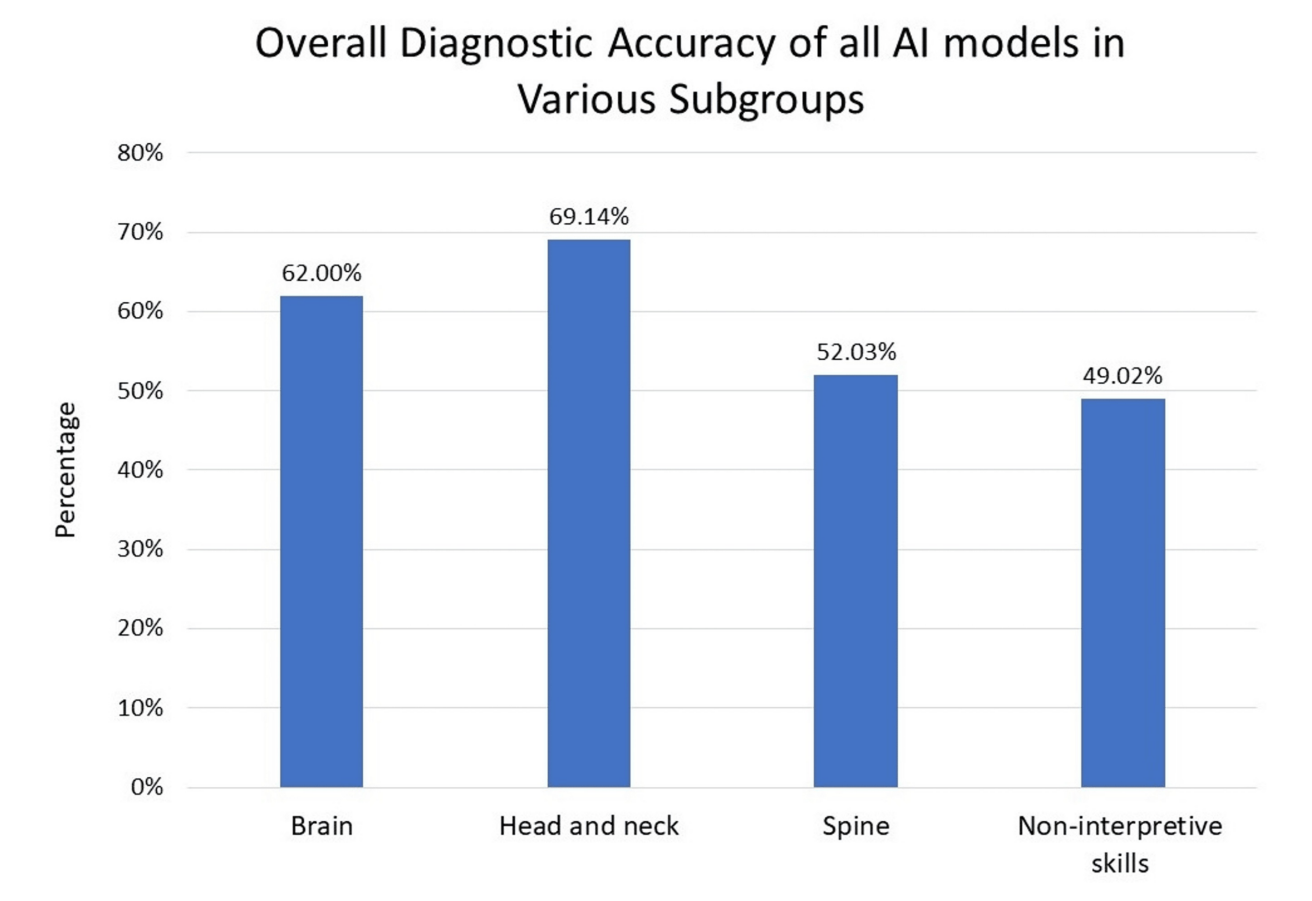
This figure displays the overall diagnostic accuracy of all AI models (ChatGPT 3.5, ChatGPT 4.0, and Google Gemini) across different neuroradiology subgroups. The combined accuracy for brain-related questions was 62%. In the head and neck category, accuracy improved to 69.14%. The accuracy was 52.03% for spine-related questions and 48.02% for non-interpretive skills.

When analyzing the diagnostic performance across different subgroups, the average accuracies for the tools were as follows: 62.00% for brain-related questions, 69.14% for head and neck, 52.03% for the spine, and 48.02% for non-interpretive skills related to the nervous system (Figure 4). These averages provide a broader perspective on the relative strengths and weaknesses of each AI tool within specific domains of neuroradiology.

In summary, ChatGPT 4.0 demonstrated the highest overall accuracy, particularly excelling in brain as well as head and neck diagnostics. Google Gemini performed best in head and neck diagnostics but lagged in other areas. ChatGPT 3.5 showed consistent performance across all categories but did not surpass the overall accuracy of ChatGPT 4.0 except for the non-interpretive questions. These findings indicate that while AI models like ChatGPT and Google Gemini have significant potential as diagnostic tools in neuroradiology, their performance varies considerably depending on the specific area of the nervous system being evaluated and how the AI model is trained. This variability underscores the importance of continuous improvement and tailored application of AI models in medical diagnostics to optimize their effectiveness and reliability across different clinical scenarios.

## Discussion

The advent of LLMs such as ChatGPT (versions 3.5 and 4.0) and Google Gemini represents a transformative development in healthcare [1-3]. These models, characterized by their advanced natural language processing capabilities, are reshaping how we approach medical diagnostics, education, and decision-making. Our study aimed to evaluate the diagnostic performance of these AI tools using neuroradiology board-style textbook questions to assess their potential utility in radiological diagnostics.

Performance analysis of AI models

Our results indicate that ChatGPT 4.0 exhibited the highest overall diagnostic accuracy at 64.89%, surpassing ChatGPT 3.5 (62.60%) and Google Gemini (55.73%). This performance highlights the advancements made with the GPT-4 architecture, particularly in handling complex radiological scenarios. The superior accuracy of ChatGPT 4.0 in diagnosing brain and head and neck conditions underscores its potential as a valuable tool in these areas. Conversely, while showing commendable performance in head and neck diagnostics, Google Gemini lagged in other categories, illustrating the variable strengths of different AI models.

ChatGPT 3.5, although slightly less accurate than its successor, demonstrated consistent performance across all diagnostic categories, reflecting its robust, albeit less advanced, capabilities. These findings align with other research indicating that GPT-4's enhancements significantly improve diagnostic precision compared to earlier models [7,8].

Subgroup performance and comparative analysis

The performance of AI tools varied significantly across different subgroups: brain, head and neck, spine, and non-interpretive skills. ChatGPT 4.0 demonstrated superior accuracy in brain and head and neck diagnostics but showed relatively lower performance in spine and non-interpretive skills. ChatGPT 3.5 excelled in non-interpretive questions, while ChatGPT 4.0 demonstrated higher performance in interpretive questions that require higher and more complex performance. ChatGPT 3.5 exhibited consistent accuracy across all subgroups, while Google Gemini performed best in the head and neck category but lagged in other areas. These findings underscore the importance of recognizing the specific strengths of each model and suggest that targeted applications are necessary depending on the clinical context. For reference, Ueda et al. reported a 54% diagnostic accuracy for ChatGPT on the "Diagnosis Please" quizzes, with a 72% accuracy specifically for head and neck questions [9]. In contrast, Suthar et al. found that ChatGPT 4.0 achieved an overall accuracy of 57.86% on the AJNR's "Case of the Month," including 67.65% accuracy in head and neck cases and 55.0% in spine cases [2]. The consistently higher accuracy in the head and neck section across all three AI models suggests that this area is particularly well-represented in their training datasets.

Evolution of AI in natural language processing

The evolution of AI, particularly in natural language processing, has fundamentally altered human-computer interactions. The introduction of advanced models like GPT-4 has opened new possibilities for applications in healthcare, offering potential improvements in diagnostic accuracy and medical education [1]. The transition from GPT-3.5 to GPT-4 demonstrates significant advancements in model performance, with GPT-4 showing considerable improvements in clinical reasoning and examination benchmarks [7].

As AI systems continue to evolve and advance in sophistication, their growing intelligence promises to revolutionize a wide array of industries. This progress is expected to bring about profound changes, significantly enhancing the quality of life for individuals across the globe. The continuous maturation of these AI technologies not only opens new possibilities for innovation but also has the potential to address complex challenges in diverse fields, ultimately leading to more efficient processes, improved outcomes, and greater overall well-being for people worldwide.

Implications for medical education and practice

The capability of AI models to handle diagnostic quizzes reflects their potential utility in medical education and practice. ChatGPT-4's ability to achieve high accuracy in clinical reasoning tests, compared to earlier models and medical students, indicates its growing role in supporting and enhancing medical training and decision-making [10,11]. However, while AI models offer promising advancements, they must be integrated thoughtfully into medical practice to ensure they complement, rather than replace, human expertise.

Ethical and societal considerations

Integrating AI in radiology and other medical fields raises several ethical and societal challenges. Biases in training data can lead to skewed diagnoses and marginalize certain patient groups. Handling patient data also poses significant privacy concerns, necessitating robust data security measures. Moreover, an over-reliance on AI could depersonalize patient care and alter trust dynamics between patients and healthcare providers. The potential for skill atrophy and job displacement among radiologists further complicates the landscape, emphasizing the need for balanced AI integration that maintains the human element of care [12].

Study limitations and future directions

This study has several limitations that must be considered. The inability of ChatGPT 3.5 and Google Gemini to process images restricted the analysis to text-based questions, potentially omitting relevant diagnostic scenarios. Additionally, relying on a single textbook's diagnostic quizzes may not fully capture the range of clinical situations encountered in practice. We did not test the reproducibility of the answers. The variability in performance across different subgroups highlights the models' specific strengths and weaknesses, which may not generalize to all clinical contexts. Small sample sizes within subgroups and the potential for human error in verifying AI-generated diagnoses could affect result accuracy. Moreover, biases in the AI models' training data and the study's temporal relevance may influence the findings. Future research should address these limitations to provide a more comprehensive evaluation of AI tools in diagnostic radiology.

## Conclusions

This study demonstrates that advanced AI models, such as ChatGPT 4.0, ChatGPT 3.5, and Google Gemini, exhibit varying levels of diagnostic accuracy in neuroradiology, with ChatGPT 4.0 showing the highest overall accuracy at 64.89% and excelling in brain and head and neck diagnostics. While these AI tools hold significant promise for enhancing diagnostic accuracy and medical education, their performance differs based on the specific area of evaluation, and Google Gemini, despite performing well in head and neck diagnostics, lagged in other categories. The findings highlight the potential of AI to improve medical practice and decision-making but underscore the necessity for continuous improvements and careful integration to address ethical concerns such as data bias and privacy. Limitations, including the exclusion of image-based questions and reliance on a single textbook, point to the need for future research to evaluate AI tools in a broader range of clinical contexts to optimize their application in patient care.
